# HLA-A, -B, -C, -DRB1, -DQB1, and -DPB1 Allele and Haplotype Frequencies of 28,927 Saudi Stem Cell Donors Typed by Next-Generation Sequencing

**DOI:** 10.3389/fimmu.2020.544768

**Published:** 2020-10-22

**Authors:** Dunia Jawdat, F. Aytül Uyar, Ahmed Alaskar, Carlheinz R. Müller, Ali Hajeer

**Affiliations:** ^1^Saudi Stem Cells Donor Registry, King Abdullah International Medical Research Center, King Saud bin Abdulaziz University for Health Sciences, Ministry of National Guard Health Affairs, Riyadh, Saudi Arabia; ^2^Department of Physiology, Istanbul Medical Faculty, Istanbul University, Istanbul, Turkey; ^3^Department of Oncology, King Abdulaziz Medical City - Ministry of National Guard Health Affairs, King Abdullah International Medical Research Center, King Saud bin Abdulaziz University for Health Sciences, Riyadh, Saudi Arabia; ^4^ZKRD Zentrales Knochenmarkspender–Register für die Bundesrepublik Deutschland, Ulm, Germany; ^5^Department of Pathology and Laboratory Medicine, King Abdulaziz Medical City - Ministry of National Guard Health Affairs, King Abdullah International Medical Research Center, King Saud bin Abdulaziz University for Health Sciences, Riyadh, Saudi Arabia

**Keywords:** bone marrow registry, Saudi Arabia, population genetics, haplotype frequencies, unrelated donors

## Abstract

Human leukocyte antigen (HLA) allele and haplotype frequency distribution varies widely between different ethnicities and geographical areas. Matching for HLA alleles is essential for successful related and unrelated stem cell transplantation. Among the Saudi population, data on HLA alleles and haplotypes are limited. A cross-sectional study was performed on 28,927 bone marrow donors. The most frequent HLA alleles were HLA-A^*^02:01:01G (20.2%), A^*^24:02:01G (7.5%); B^*^51:01:01G (19.0%), B^*^50:01:01G (12.3%); C^*^06:02:01G (16.7%), C^*^07:02:01G (12.2%); DRB1^*^07:01:01 (15.7%), DRB1^*^03:01:01G (13.3%); DQB1^*^02:01:01G (29.9%), DQB1^*^03:02:01G (13.2%); and DPB1^*^04:01:01G (35.2%), DPB1^*^02:01:02G (21.8%). The most frequent HLA-A~C~B~DRB1~DQB1 haplotypes were A^*^02:01:01G~C^*^06:02:01G~B^*^50:01:01G~DRB1^*^07:01:01G~DQB1^*^02:01:01G (1.9%) and A^*^02:05:01G~C^*^06:02:01G~B^*^50:01:01G~DRB1^*^07:01:01G~DQB1^*^02:01:01G (1.6%). The most frequent HLA-A~C~B~DRB1~DQB1~DPB1 haplotypes were A^*^02:01:01G~C^*^15:02:01G~B^*^51:01:01G~DRB1^*^04:02~DQB1^*^03:02:01G~DPB1^*^04:01:0G (1%) and A^*^02:01:01G~C^*^07:02:01G~B^*^07:02:01G~DRB1^*^15:01:01G~DQB1^*^06:02:01G~ DPB1^*^04:01:01G (0.9%). Based on these haplotype frequencies, we provide forecasts for the fraction of patients with full matching and single mismatched donors for 3 to 6 loci depending on the registry size. With one million donors, about 50% of the patients would find an 8/8 match and 90% a 7/8 match. These data are essential for registry planning, finding unrelated stem cell donors, population genetic studies, and HLA disease associations.

## Introduction

The human leukocyte antigen (HLA) system is encoded by the most polymorphic genes known, located on the short arm of chromosome 6 (1). The HLA system is divided into class I (HLA-A, -B, and -C) and class II (HLA-DR, -DQ, and -DP) loci. Recent advances in sequencing technologies helped in unraveling a vast number of new HLA alleles. So far, 27,589 different HLA allele sequences have been reported based on the latest IPD-IMGT/HLA Database 3.41.0 (2). The HLA system plays an essential role in activating the immune system through the presentation of processed antigens to CD4+ and CD8+ T cells. On the other hand, HLA is a key player in the success of organ and stem cell transplantation. Mismatch in both HLA class I and II alleles between donor and recipient is a major cause of organ rejection, graft failure, and graft-vs. host disease following hematopoietic stem transplantation (3). In addition, certain HLA alleles were found to be associated with autoimmune diseases, such as ankylosing spondylitis, Type I diabetes, Behçet's disease, celiac disease, and rheumatoid arthritis (4).

Saudi society has a very high rate (more than 70%) of consanguineous marriages (5). Together with having large families, the chances of finding an HLA-matched relative are very high. However, a proportion (30–40%) of patients cannot find a matched donor (6). Therefore, the Saudi Stem Cell donor registry (SSCDR) was launched in 2011 in Riyadh, Saudi Arabia. SSCDR is a national organization that manages unrelated stem cell donors and cord blood units as a source for allogeneic stem cell transplantation. The aim of SSCDR establishment was to provide stem cells for those patients without a matching family donor. Today, SSCDR has more than 75,000 registered donors who are willing to donate their stem cells for any patient in need worldwide. Currently, SSCDR works closely with 14 donor recruitment centers in Saudi Arabia and has facilitated more than 67 successful stem cell transplants, 40 for national patients and 27 for international patients, including from Germany, the United Kingdom, the United States, Spain, Italy, Turkey, Sweden, Norway, India, and Australia. The goal of any stem cell registry is to reflect the HLA allele polymorphism of the population it serves. This can be challenging in countries such as Saudi Arabia with high HLA diversity due to a large number of ethnic groups from different countries that have settled there over the years in the holy cities of Mecca and Madinah.

The aim of this study was to evaluate the HLA class I and class II alleles and haplotypes from the existing database of registered Saudi stem cell donors. This study is essential for stem cell transplantation programs as the level of matching between donor and recipient is very important for the outcome of hematopoietic stem cell transplantation.

## Materials and Methods

### Population

A cross-sectional study was performed on 28,927 Saudi volunteers for bone marrow donors registered in the database of the SSCDR and typed for HLA-A, -B, -C, -DRB, -DQB1, and -DPB1 at a high-resolution level from 2013 to 2017. All donors included in the analysis fulfilled the eligibility criteria and were unrelated Saudi Arabs, 49% males and 51% females, 18–60 years old with an average age of 31 from different regions within the country, including Riyadh, Qasim, Dammam, Assire, Albaha, Jizan, Jeddah, and Madinah. These regions were not linked directly to each subject at that time; therefore, this cohort was not classified or grouped geographically.

### HLA Class I and HLA Class II Typing

This project was approved by the local IRB at King Abdulaziz Medical City, Riyadh, National Guard Health Affair, Saudi Arabia. All donors were asked to sign a formal written consent. Blood samples, six drops on filter paper were collected. Total genomic DNA was extracted using QIAamp96 DNA Blood Mini Kit according to the manufacturer's instructions (Qiagen, Hilden, Germany). HLA-A, -B, -C, -DRB1, -DQB1, and -DPB1 genotyping was carried out by next-generation sequencing (NGS) at the laboratories of Histogenetics (Ossining, NY). Briefly, HLA alleles were typed using high-resolution (Class I: Exon 2 and 3, Class II: Exon 2) (7) using the Illumina platform (San Diego, CA). The sequencing templates (DNA libraries) for Illumina were prepared according to the manufacturer's protocols, involving two rounds of amplification. For sequence data analysis, MiSeq generates FastQ files that are used by the in-house NGSAutoTyper software to perform the final typing. HistoS software is used for further review and analysis, and the data is then transferred for final reporting to HistoTyper software. For certain typing results, we gave alternative combinations with G codes. Then, we selected the highest probable combination based on the frequency.

Homozygous typing results were controlled by the following method. For class I, exon 2 and 3 are repeated with alternative primers complementary to sequences in the middle of exon 2 and exon 3 to generate amplicons spanning mid exon 2–intron 2–exon 3 (bridge amplification). In addition known HLA-B–C linkages are checked. If the bridge amplicon or B–C association confirms the homozygosity, then results are reported. If the results indicate heterozygous typing, a 1-KB amplicon is repeated spanning the whole exon 2–intron 2–Exon 3 with PacBio sequencing technology. For class II, DRB1 is analyzed with one generic and two group-specific amplifications. DQB1 and DPB1 are analyzed with one generic and one group-specific amplification. In addition, known DRB1–DRB3/4/5 and DRB1–DQB1 linkages are checked. In the presence of base occurrence more than 300 and a known linkage, homozygous results are accepted. In the absence of the latter, sequencing is repeated by a long-range amplicon spanning exon 2–intron 2–exon 3 using PacBio technology.

### Criteria for Common and Well-Documented (CWD) Alleles

In this study, the CWD alleles were defined according to CIWD 3.0.0 inclusion criteria (8). Common alleles are those with ≥1 in 10,000, and well-documented alleles are those with ≥5 occurrences. Intermediate alleles (>1 in 100,000) were not calculated due to low sample size.

### Statistical Analysis

The population analyses and genetic diversity measures were calculated using Arlequin 3.5 software (9). In particular, allele frequencies and three, five, and six locus haplotype frequencies were determined by the expectation-maximization (EM) algorithm (10).

A pairwise linkage disequilibrium (LD) test was performed with a Markov chain algorithm using estimated haplotypes for each individual obtained with the Excoffier–Laval–Balding (ELB) algorithm. Frequencies of common haplotypes were similar with both methods, and those with significant pairwise LD are shown in the tables. For testing the deviation from Hardy Weinberg equilibrium, a Markov chain algorithm was performed. A selective neutrality test could not be performed due to software allele and haplotype number limitations. Computed two loci (HLA-B-C and HLA-DRB1-DQB1) associations were listed as common (with observed frequency of more than 100 times) or rare (with observed frequency of <100 times).

The probability of finding a completely matching and single mismatched donor for 3–6 loci, depending on the registry size, was calculated based on the formula first shown in 1989 (11) and refined in 2014 (12). As is a common practice today (13), matching was based on the antigen recognition domain (exons 2 and 3 for class I and exon 2 only for class II).

Data used in this analysis are available in the Allele Frequencies Net Database (http://allelefrequencies.net/hla6006a.asp?hla_population=3685) and on a public website (https://www.ihiw18.org/).

## Results

### HLA Allele Frequencies

A total of 103 HLA-A alleles, 154 HLA-B alleles, 76 HLA-C alleles, 96 HLA-DRB1 alleles, 37 HLA-DQB1 alleles, and 48 HLA-DPB1 alleles were found in our cohort. Table 1 shows the allele frequency for HLA-A, -B, -C, -DRB1, -DQB1, and -DPB1. For HLA class I, the following alleles were the most frequent alleles in HLA-A, -B, and -C, respectively:A^*^02:01:01G (20.2%), A^*^24:02:01G (7.5%), A^*^01:01:01G (7.1%), A^*^68:01:01G (6.3%), and A^*^03:01:01G (5.9); B^*^51:01:01G (19.0%), B^*^50:01:01G (12.3%), B^*^08:01:01G (6.9%), B^*^07:02:01G (5.0%), and B^*^53:01:01G (3.9%); C^*^06:02:01G (16.7%), C^*^07:02:01G (12.2%), C^*^04:01:01G (12.2%), C^*^15:02:01G (10.7%), and C^*^07:01:01G (9.7%). For HLA class II, the following alleles were the most frequent alleles in HLA-DRB1, -DQB1, and -DPB1, respectively:DRB1^*^07:01:01(15.7%), DRB1^*^03:01:01G (13.3%), DRB1^*^13:02:01G (6.7%), DRB1^*^15:01:01 (6.3%), and DRB1^*^13:01:01G (6.2%); DQB1^*^02:01:01G (29.9%), DQB1^*^03:02:01G (13.2%), DQB1^*^03:01:01G (12.1%), DQB1^*^05:01:01G (9.9%), and DQB1^*^06:03:01G (6.9%); DPB1^*^04:01:01G (35.2%), DPB1^*^02:01:02G (21.8%), DPB1^*^03:01:01G (11.6%), DPB1^*^04:02:01G (6.5%), and DPB1^*^17:01:01G (5.4%). All subjects were typed successfully. Four new alleles were identified in this Saudi cohort (HLA-A^*^02:433, HLA-A^*^02:434, HLA-C^*^14:02:13, and HLA-DRB1^*^14:145); the data are available on Allele frequency and published (14–16). Table 2 shows the results of the Hardy Weinberg equilibrium analysis. Heterozygosity in all six loci was observed in this large cohort significantly less than what is expected by chance.

**Table 1:** HLA-A, HLA-B, HLA-C, HLA-DRB1, HLA-DQB1, and HLA-DPB1 allele frequencies in the Saudi Stem cell donors registry.

**HLA-A**	**Frequency**	**HLA-B**	**Frequency**	B*57:01:07	**0.000052**	C*06:27	**0.000052**	DRB1*08:20	**0.000017**	DPB1*46:01	**0.000052**
A*02:01:01G	0.201991	B*51:01:01G	0.190117	B*07:14	0.000035	C*07:29	0.000052	DRB1*10:13	0.000017	DPB1*72:01	0.000052
A*24:02:01G	0.074740	B*50:01:01G	0.123933	B*08:38	0.000035	C*06:49N	0.000035	DRB1*11:01:27	0.000017	DPB1*75:01	0.000052
A*01:01:01G	0.071214	B*08:01:01G	0.069036	B*15:06	0.000035	C*07:51	0.000035	DRB1*11:14	0.000017	DPB1*88:01	0.000052
A*68:01:01G	0.063211	B*07:02:01G	0.050385	B*15:12:01G	0.000035	C*01:03:01G	0.000017	DRB1*11:16	0.000017	DPB1*233:01	0.000035
A*03:01:01G	0.059028	B*53:01:01G	0.038960	B*15:229	0.000035	C*02:14:01G	0.000017	DRB1*11:36	0.000017	DPB1*290:01	0.000035
A*31:01:02G	0.052771	B*41:01	0.034449	B*15:47	0.000035	C*02:26	0.000017	DRB1*11:37	0.000017	DPB1*85:01	0.000035
A*26:01:01G	0.052753	B*58:01:01G	0.033775	B*15:73	0.000035	C*02:43	0.000017	DRB1*13:11	0.000017	DPB1*02:01:07	0.000017
A*23:01:01G	0.044923	B*35:01:01G	0.027898	B*35:30	0.000035	C*04:42	0.000017	DRB1*13:178	0.000017	DPB1*09:01:02	0.000017
A*02:05:01G	0.044284	B*18:01:01G	0.027362	B*35:34	0.000035	C*04:46	0.000017	DRB1*13:49	0.000017	DPB1*132:01	0.000017
A*32:01:01G	0.041173	B*49:01:01G	0.024838	B*35:38	0.000035	C*06:06	0.000017	DRB1*13:66	0.000017	DPB1*137:01	0.000017
A*33:03:01G	0.035728	B*52:01	0.023991	B*44:06	0.000035	C*06:127	0.000017	DRB1*14:145	0.000017	DPB1*20:01	0.000017
A*11:01:01G	0.035002	B*35:03:01G	0.021209	B*51:21	0.000035	C*07:02:36	0.000017	DRB1*14:158	0.000017	DPB1*214:01	0.000017
A*30:02:01G	0.032081	B*14:02	0.020932	B*51:29	0.000035	C*07:02:52	0.000017	DRB1*14:33	0.000017	DPB1*25:01	0.000017
A*30:01:01G	0.025651	B*15:17:01G	0.020673	B*82:01	0.000035	C*07:182	0.000017	DRB1*14:38	0.000017	DPB1*266:01	0.000017
A*68:02:01G	0.016593	B*35:08	0.019532	B*08:03	0.000017	C*07:19	0.000017	DRB1*14:68	0.000017	DPB1*27:01	0.000017
A*29:01:01G	0.016300	B*13:02:01G	0.016040	B*13:03	0.000017	C*07:27	0.000017	DRB1*14:69	0.000017	DPB1*423:01	0.000017
A*03:02:01G	0.015729	B*38:01	0.015816	B*13:11	0.000017	C*08:28	0.000017	**HLA- DQB1**	**Frequency**	DPB1*50:01	0.000017
A*02:02:01G	0.010959	B*15:03:01G	0.013845	B*15:07:01G	0.000017	C*12:101	0.000017	DQB1*02:01:01G	0.298994	DPB1*523:01	0.000017
A*30:04:01G	0.010129	B*07:05:01G	0.013569	B*15:156	0.000017	C*14:02:02	0.000017	DQB1*03:02:01G	0.132039	DPB1*526:01	0.000017
A*33:01:01G	0.010043	B*44:02:01G	0.011304	B*15:38	0.000017	C*15:22	0.000017	DQB1*03:01:01G	0.120493	DPB1*54:01	0.000017
A*29:02:01G	0.009749	B*37:01:01G	0.011114	B*15:67	0.000017	C*15:62	0.000017	DQB1*05:01:01G	0.098662	DPB1*641:01	0.000017
A*68:01:02G	0.008677	B*35:02:01G	0.010786	B*18:06	0.000017	C*17:28	0.000017	DQB1*06:03:01G	0.068811	DPB1*643:01	0.000017
A*74:01:01G	0.007363	B*44:03:01G	0.010371	B*18:109	0.000017	C*18:06	0.000017	DQB1*06:02:01G	0.067204	DPB1*67:01	0.000017
A*66:01:01G	0.007277	B*55:01:01G	0.009731	B*18:26	0.000017	**HLA-DRB1**	**Frequency**	DQB1*05:02:01G	0.052391	DPB1*89:01	0.000017
A*74:03	0.006344	B*39:24	0.008781	B*18:73	0.000017	DRB1*07:01:01G	0.156930	DQB1*06:04:01G	0.044491	DPB1*94:01	0.000017
A*01:03	0.006084	B*42:01	0.008418	B*27:08	0.000017	DRB1*03:01:01G	0.133889	DQB1*06:01:01G	0.030404		
A*24:03:01G	0.004304	B*40:01:01G	0.008331	B*27:09	0.000017	DRB1*13:02:01G	0.067325	DQB1*04:02:01G	0.021900		
A*02:11:01G	0.003734	B*40:06:01G	0.008158	B*27:26	0.000017	DRB1*15:01:01G	0.063245	DQB1*06:09:01G	0.021174		
A*34:02	0.003682	B*45:01:01G	0.007795	B*27:61	0.000017	DRB1*13:01:01G	0.061517	DQB1*03:03:02G	0.020016		
A*69:01	0.003198	B*39:10	0.007536	B*35:13	0.000017	DRB1*04:03:01G	0.059616	DQB1*05:03:01G	0.016144		
A*24:07	0.002385	B*73:01	0.007502	B*35:158	0.000017	DRB1*04:05	0.053186	DQB1*03:05:01G	0.002904		
A*02:22:01G	0.002161	B*57:01:01G	0.007121	B*35:21	0.000017	DRB1*10:01:01G	0.047291	DQB1*05:04	0.002575		
A*02:06:01G	0.001970	B*57:03:01G	0.006240	B*37:02	0.000017	DRB1*11:01:01G	0.035901	DQB1*04:01:01G	0.000346		
A*26:17	0.001936	B*15:08	0.005877	B*37:03N	0.000017	DRB1*04:02	0.034812	DQB1*06:08	0.000311		
A*02:03:01G	0.001746	B*44:03:02G	0.005790	B*39:05	0.000017	DRB1*16:01	0.029730	DQB1*03:04:01G	0.000190		
A*36:01	0.001711	B*15:10	0.005479	B*39:31	0.000017	DRB1*01:02	0.029713	DQB1*03:01:27	0.000173		
A*31:04	0.001175	B*41:02	0.004840	B*39:99	0.000017	DRB1*11:04:01G	0.028399	DQB1*06:31	0.000156		
A*30:10	0.001020	B*27:05:02G	0.004788	B*40:10	0.000017	DRB1*15:02:01G	0.026722	DQB1*06:126	0.000104		
A*02:85	0.000847	B*51:08	0.004580	B*40:23	0.000017	DRB1*15:03:01G	0.016179	DQB1*06:153	0.000104		
A*24:02:05	0.000830	B*15:01:01G	0.003820	B*40:295	0.000017	DRB1*01:01:01G	0.015608	DQB1*06:123	0.000086		
A*80:01:01G	0.000812	B*58:02	0.003630	B*44:07	0.000017	DRB1*08:04	0.013534	DQB1*06:11	0.000069		
A*02:17:01G	0.000778	B*27:02	0.003526	B*44:10	0.000017	DRB1*11:02	0.011702	DQB1*02:74	0.000035		
A*25:01:01G	0.000709	B*57:02	0.003077	B*44:15	0.000017	DRB1*16:02:01G	0.011494	DQB1*05:05	0.000035		
A*01:02	0.000622	B*40:02:01G	0.002990	B*44:197	0.000017	DRB1*04:01:01G	0.011408	DQB1*03:02:03	0.000017		
A*02:14	0.000536	B*15:02:01G	0.002973	B*44:29	0.000017	DRB1*14:01:01G	0.010889	DQB1*03:146	0.000017		
A*02:08	0.000398	B*51:02	0.002869	B*51:01:02	0.000017	DRB1*11:01:02	0.010613	DQB1*03:229	0.000017		
A*02:93	0.000363	B*27:03	0.002593	B*51:01:04	0.000017	DRB1*13:03:01G	0.010406	DQB1*03:232	0.000017		
A*24:17	0.000363	B*13:01:01G	0.002333	B*51:208	0.000017	DRB1*03:02	0.009005	DQB1*05:147	0.000017		
A*34:01	0.000363	B*47:01:01G	0.002126	B*52:04	0.000017	DRB1*04:04	0.006897	DQB1*05:79	0.000017		
A*26:12	0.000294	B*42:02:01G	0.002091	B*78:02	0.000017	DRB1*12:02	0.006153	DQB1*06:01:08	0.000017		
A*02:01:09	0.000277	B*14:01	0.001970	**HLA-C**	**Frequency**	DRB1*04:06:01G	0.006032	DQB1*06:22:01	0.000017		
A*02:07:01G	0.000277	B*47:03	0.001780	C*06:02:01G	0.166713	DRB1*09:01:02G	0.005064	DQB1*06:22:03	0.000017		
A*02:01:04	0.000259	B*38:02:01G	0.001728	C*07:02:01G	0.122256	DRB1*12:01:01G	0.004511	DQB1*06:32	0.000017		
A*24:10	0.000259	B*39:01:01G	0.001694	C*04:01:01G	0.121461	DRB1*14:04	0.004114	DQB1*06:89	0.000017		
A*02:16:01G	0.000242	B*81:01:01G	0.001625	C*15:02:01G	0.107270	DRB1*04:08	0.002230	**HLA- DPB1**	**Frequency**		
A*02:433	0.000242	B*15:18:01G	0.001366	C*07:01:01G	0.096605	DRB1*08:03	0.002005	DPB1*04:01:01G	0.351851		
A*11:03:01G	0.000225	B*56:01:01G	0.001366	C*17:01:01G	0.046203	DRB1*15:06	0.001832	DPB1*02:01:02G	0.217807		
A*24:02:04	0.000207	B*15:16	0.000968	C*12:03:01G	0.042763	DRB1*11:12	0.001504	DPB1*03:01:01G	0.115705		
A*02:20	0.000190	B*27:07:01G	0.000968	C*03:02:01G	0.028157	DRB1*13:05	0.001279	DPB1*04:02:01G	0.065043		
A*02:01:05	0.000138	B*40:12	0.000882	C*16:02:01G	0.027552	DRB1*14:06	0.001124	DPB1*17:01:01G	0.053877		
A*02:434	0.000121	B*35:05	0.000864	C*14:02:01G	0.024856	DRB1*15:02:02	0.001089	DPB1*14:01:01G	0.046531		
A*29:10	0.000121	B*15:13	0.000726	C*12:02:01G	0.024199	DRB1*04:07:01G	0.000951	DPB1*13:01:01G	0.044180		
A*30:08	0.000121	B*39:06	0.000691	C*08:02:01G	0.023248	DRB1*08:01:01G	0.000847	DPB1*01:01:01G	0.029816		
A*30:09	0.000121	B*82:02	0.000622	C*15:05:01G	0.020967	DRB1*11:03	0.000657	DPB1*10:01:01G	0.010578		
A*66:03	0.000121	B*15:31	0.000605	C*02:02:02G	0.017198	DRB1*03:01:02	0.000363	DPB1*01:01:02G	0.009749		
A*01:23	0.000104	B*15:25:01G	0.000570	C*15:04:01G	0.017112	DRB1*11:06:01G	0.000328	DPB1*05:01:01G	0.007415		
A*29:11	0.000104	B*51:07	0.000519	C*16:04	0.015522	DRB1*14:07	0.000311	DPB1*15:01:01G	0.007225		
A*11:02:01G	0.000086	B*48:01:01G	0.000501	C*01:02:01G	0.015003	DRB1*08:02	0.000294	DPB1*09:01:01	0.007156		
A*66:02	0.000086	B*46:01:01G	0.000484	C*16:01:01G	0.013309	DRB1*08:06	0.000277	DPB1*11:01:01	0.005721		
A*01:06	0.000069	B*18:02	0.000398	C*05:01:01G	0.013223	DRB1*14:05	0.000242	DPB1*30:01	0.004719		
A*31:16	0.000069	B*14:03	0.000346	C*03:04:01G	0.009230	DRB1*11:04:02	0.000207	DPB1*26:01	0.002869		
A*29:12	0.000052	B*15:29	0.000346	C*07:04:01G	0.008262	DRB1*15:01:16	0.000207	DPB1*45:01	0.002662		
A*29:15	0.000052	B*44:05	0.000346	C*18:01:01G	0.006153	DRB1*13:02:05	0.000190	DPB1*18:01	0.002282		
A*34:05	0.000052	B*15:32	0.000328	C*03:03:01G	0.006067	DRB1*04:10:01G	0.000156	DPB1*55:01:01G	0.001988		
A*02:392	0.000035	B*27:06	0.000311	C*02:10:01G	0.005566	DRB1*16:05	0.000156	DPB1*23:01:01G	0.001849		
A*02:42	0.000035	B*78:01	0.000311	C*03:04:02	0.005030	DRB1*13:04	0.000121	DPB1*28:01:01G	0.001677		
A*03:202	0.000035	B*50:02	0.000294	C*08:01:01G	0.004131	DRB1*14:15	0.000121	DPB1*39:01:01G	0.001262		
A*11:04	0.000035	B*15:05	0.000277	C*14:03	0.002627	DRB1*15:04	0.000121	DPB1*19:01:01G	0.001158		
A*23:30	0.000035	B*51:05	0.000277	C*04:03	0.002454	DRB1*03:06	0.000104	DPB1*34:01	0.001003		
A*26:07	0.000035	B*15:21	0.000207	C*04:07	0.002247	DRB1*08:08	0.000104	DPB1*47:01:01G	0.000899		
A*26:30	0.000035	B*54:01:01G	0.000207	C*04:04	0.000778	DRB1*11:11:01G	0.000104	DPB1*02:02:01G	0.000726		
A*30:103	0.000035	B*15:09	0.000190	C*07:05	0.000380	DRB1*11:08	0.000086	DPB1*16:01	0.000691		
A*31:06	0.000035	B*51:06	0.000190	C*12:05	0.000380	DRB1*11:15	0.000086	DPB1*21:01	0.000311		
A*43:01	0.000035	B*15:123:01G	0.000173	C*08:04	0.000363	DRB1*14:02	0.000086	DPB1*06:01:01G	0.000277		
A*01:09	0.000017	B*27:04:01G	0.000173	C*07:26	0.000311	DRB1*07:03	0.000069	DPB1*02:01:18	0.000242		
A*01:106	0.000017	B*40:16	0.000173	C*16:46	0.000259	DRB1*14:17	0.000069	DPB1*40:01	0.000190		
A*01:91	0.000017	B*51:09	0.000156	C*04:06	0.000156	DRB1*13:16	0.000052	DPB1*91:01:01G	0.000173		
A*02:64	0.000017	B*15:55	0.000121	C*08:03:01G	0.000156	DRB1*03:01:20	0.000035	DPB1*312:01	0.000156		
A*03:08	0.000017	B*15:71	0.000121	C*03:19	0.000138	DRB1*04:13	0.000035	DPB1*325:01	0.000156		
A*03:09	0.000017	B*15:35	0.000104	C*12:139	0.000121	DRB1*04:18	0.000035	DPB1*80:01	0.000156		
A*24:02:19	0.000017	B*40:50	0.000104	C*15:07	0.000121	DRB1*10:01:08	0.000035	DPB1*02:01:04	0.000138		
A*24:02:48	0.000017	B*55:02:01G	0.000104	C*04:10	0.000104	DRB1*13:03:02	0.000035	DPB1*29:01	0.000138		
A*24:06	0.000017	B*56:04	0.000104	C*15:30	0.000104	DRB1*13:14	0.000035	DPB1*422:01	0.000138		
A*24:256	0.000017	B*15:24	0.000086	C*01:17	0.000086	DRB1*13:18	0.000035	DPB1*49:01:01G	0.000138		
A*24:312N	0.000017	B*57:04	0.000086	C*03:16	0.000086	DRB1*13:19	0.000035	DPB1*81:01	0.000121		
A*25:11	0.000017	B*07:10	0.000069	C*07:13	0.000086	DRB1*14:03	0.000035	DPB1*93:01	0.000104		
A*26:08	0.000017	B*48:05	0.000069	C*14:02:03	0.000086	DRB1*14:10	0.000035	DPB1*63:01	0.000086		
A*30:25	0.000017	B*15:11:01G	0.000052	C*15:06	0.000086	DRB1*03:01:16	0.000017	DPB1*634:01	0.000086		
A*30:29	0.000017	B*15:37	0.000052	C*04:132	0.000069	DRB1*03:15	0.000017	DPB1*71:01	0.000086		
A*30:76N	0.000017	B*15:64	0.000052	C*12:04	0.000069	DRB1*03:25	0.000017	DPB1*57:01:01G	0.000069		
A*68:13	0.000017	B*15:75	0.000052	C*03:88	0.000052	DRB1*03:58	0.000017	DPB1*31:01	0.000052		
A*68:38	0.000017	B*18:03	0.000052	C*03:94	0.000052	DRB1*04:101	0.000017	DPB1*35:01	0.000052		
A*74:16	0.000017	B*39:15	0.000052	C*05:18	0.000052	DRB1*04:15	0.000017	DPB1*41:01	0.000052		

**Table 2:** Hardy Weinberg equilibrium.

**Locus**	**Genotype**	**Observed heterozygosity**	**Expected heterozygosity**	***P***
HLA-A	28927	0.886	0.924	<0.001
HLA-C	28927	0.876	0.912	0.005
HLA-B	28927	0.887	0.931	0.008
HLA-DRB1	28927	0.886	0.929	0.001
HLA-DQB1	28927	0.810	0.852	<0.001
HLA-DPB1	28927	0.770	0.803	<0.001

**Figure 1:**
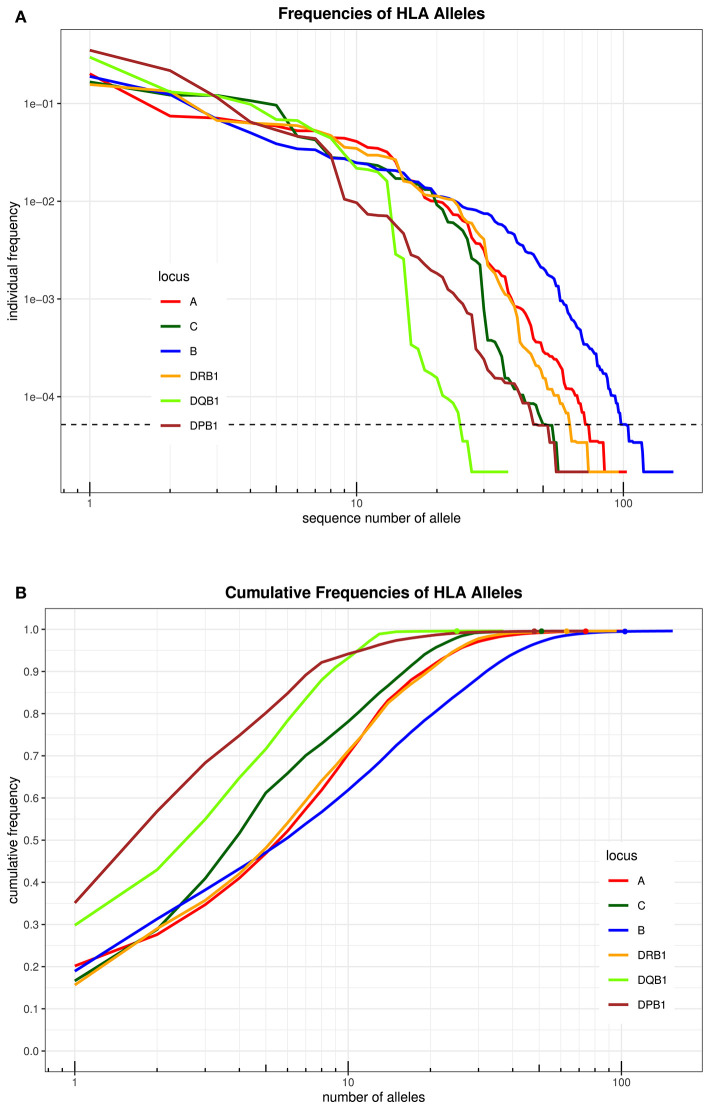
**(A)** The alleles of each locus are ordered by descending frequency on the x-axis, and the curve shows their frequency. Both axes are in logarithmic scale. The dotted horizontal line shows the frequency corresponding to three copies in the sample. **(B)** For each number (*n*) on the x-axis in logarithmic scale, the curve depicts the cumulative frequency of the set of the *n* most frequent alleles of each locus. The closer a curve is to the left and top, the more homogeneous a population is for this locus. The dots on each curve mark the cumulative frequency of all alleles seen at least three times in the sample.

**Figure 2:**
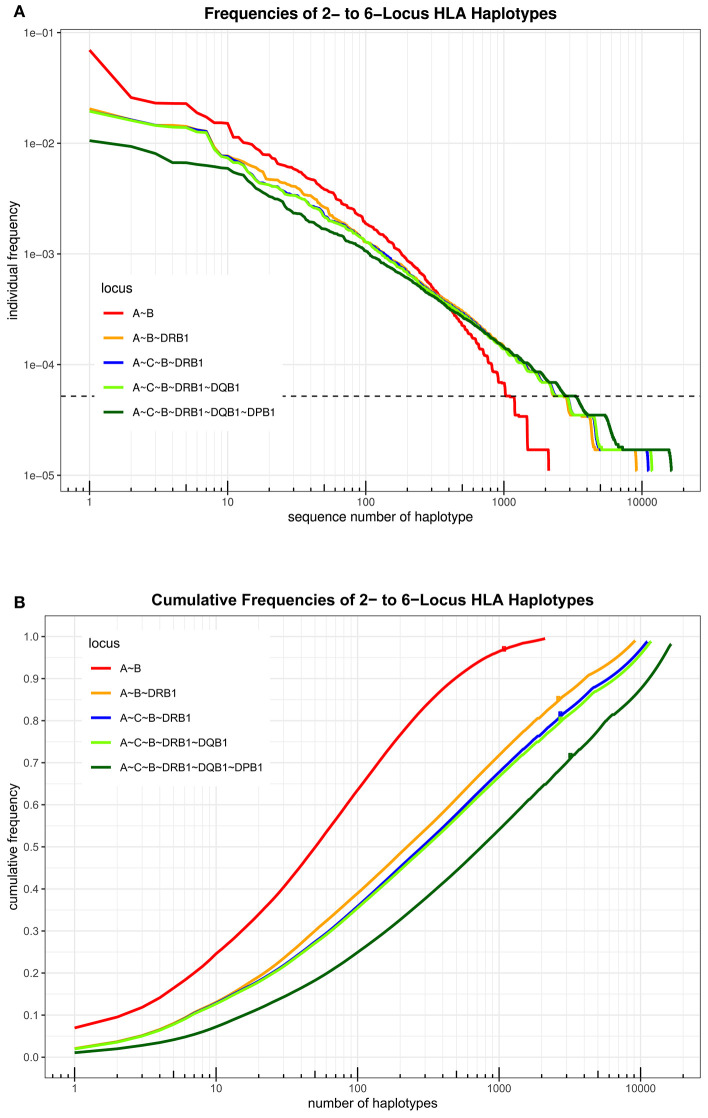
**(A)** The haplotypes of each set of loci are ordered by descending frequency on the x-axis, and the curve shows their frequency. Both axes are in logarithmic scale. The dotted horizontal line shows the frequency corresponding to three copies in the sample. **(B)** For each number *n* on the x-axis in logarithmic scale, the curve depicts the cumulative frequency of the set of the *n* most frequent haplotypes of each locus combination. The dots on each curve mark the cumulative frequency of all haplotypes seen at least three times in the sample.

An overview over the frequency distribution of all six loci is shown in Table 3 and Figures 1A,B. Apparently, HLA-DQB1 and HLA-DPB1 are much less polymorphic than the other loci, in particular due to a number of frequent alleles. HLA-A and HLA-DRB1 carry a very similar amount of information that is higher than in HLA-C but lower than in HLA-B, which is by far the most polymorphic of the six loci. As is well-known, a rare allele has to be seen at least three times in order to be more frequent than an unobserved one significantly with *p* < 0.05 according to the Poisson distribution. As a consequence, we have marked this frequency in Figure 1A and the corresponding cumulative frequency in Figure 1B. Figures 2A,B present two analogous plots for the haplotypes of 2–6 locus combinations frequently considered in donor–patient matching, and other combinations are shown in Supplementary Tables 1–3 and Supplementary Figures 1A,B, 2A,B. These figures demonstrate that our study covers more than 95% of all 2-locus haplotypes of the Saudi population; however, this fraction diminishes to about 80% for 4 and 5 loci and 70% for 6 loci.

**Table 3:** Individual cumulated frequency distribution of all six loci.

	**Locus**	**Shannon's entropy**	**Alleles required to cover**				
			**50%**	**75%**	**90%**	**95%**	**total**
1	A	4.36	6	12	19	25	103
2	C	4.02	4	9	17	21	76
3	B	4.81	6	17	33	43	154
4	DRB1	4.36	6	12	20	25	96
5	DQB1	3.22	3	6	9	11	37
6	DPB1	2.99	2	5	8	12	74

*For each of the six loci, the table shows Shannon's entropy of the frequency distribution as well as the number of the most frequent alleles required to cover 50, 75, 90, and 95% of the gene pool as well as the total number of alleles observed in the sample*.

The pairwise linkage disequilibrium (LD) parameters, frequency, observed, expected, D, D', and *p* values for each possible pair of two HLA alleles are estimated in Supplementary Table 4. Most alleles at HLA-A and HLA-B are in strong LD with HLA-C. The relative strength of LD between two HLA loci was calculated based on the pairwise LD parameters for all the allelic pairs. Common and rare associations between HLA-DRB1-DQB1 and HLA-B-C in the Saudi population are presented in Tables 4, 5, respectively. CWD alleles are presented in Table 6. CWD alleles represent 60% of the allelic distribution in the Saudi population. The data used in this analysis are available in the Allele Frequencies Net Database (http://allelefrequencies.net/hla6006a.asp?hla_population=3685) and on a public website (https://www.ihiw18.org/).

**Table 4:** Common and rare HLA-DRB1-DQB1 associations in the Saudi population.

**HLA-DRB1**	**HLA-DQB1**			
	**Common**		**Rare**	
DRB1*01:01	DQB1*05:01	DQB1*05:04	DQB1*05:03	–
DRB1*01:02	DQB1*05:01	–	DQB1*06:02	–
DRB1*03:01	DQB1*02:01	DQB1*05:02	DQB1*05:01	DQB1*03:01
DRB1*03:02	DQB1*04:02	–	–	–
DRB1*04:01	DQB1*03:02	DQB1*03:01	DQB1*02:01	–
DRB1*04:02	DQB1*03:02	–	DQB1*03:01	DQB1*02:01
DRB1*04:03	DQB1*03:02	DQB1*03:05	DQB1*03:01	DQB1*02:01
DRB1*04:04	DQB1*03:02	–	–	–
DRB1*04:05	DQB1*03:02	DQB1*02:01	DQB1*04:01	DQB1*04:02
DRB1*07:01	DQB1*02:01	DQB1*03:03	DQB1*03:02	DQB1*03:01
DRB1*08:01	DQB1*04:02	–	–	–
DRB1*08:02	DQB1*04:02	–	DQB1*03:01	DQB1*03:02
DRB1*08:03	DQB1*03:01	–	DQB1*06:01	–
DRB1*08:04	DQB1*03:01	–	DQB1*06:02	–
DRB1*08:06	DQB1*03:01	DQB1*04:02	DQB1*05:02	DQB1*02:01
DRB1*09:01	DQB1*03:03	DQB1*02:01	DQB1*03:02	DQB1*03:01
DRB1*10:01	DQB1*05:01	–	DQB1*06:02	–
DRB1*11:01	DQB1*03:01	DQB1*05:02	DQB1*05:01	DQB1*06:02
DRB1*11:02	DQB1*03:01	–	DQB1*05:01	DQB1*02:01
DRB1*11:04	DQB1*03:01	DQB1*06:03	DQB1*05:02	DQB1*02:01
DRB1*13:01	DQB1*06:03	DQB1*05:01	DQB1*06:04	DQB1*06:02
DRB1*13:02	DQB1*06:04	DQB1*06:09	DQB1*06:03	DQB1*05:01
DRB1*13:03	DQB1*03:01	–	DQB1*02:01	DQB1*03:02
DRB1*14:01	DQB1*05:03	–	DQB1*05:02	DQB1*05:01
DRB1*15:01	DQB1*06:02	DQB1*06:01	DQB1*05:02	DQB1*06:03
DRB1*15:02	DQB1*06:01	DQB1*05:01	DQB1*06:01	DQB1*05:02
DRB1*15:03	DQB1*06:02	DQB1*06:03	DQB1*05:01	–
DRB1*16:01	DQB1*05:02	–	DQB1*06:02	DQB1*06:04
DRB1*16:02	DQB1*05:02	–	DQB1*06:03	–

**Table 5:** Common and rare HLA-B-C associations in the Saudi population.

**HLA-B**	**HLA-C**			
		**Common**	**Rare**	
B*51:01	C*15:02	C*14:02	C*03:02	C*06:02
B*50:01	C*06:02	C*07:01	C*12:03	C*15:02
B*08:01	C*07:02	C*07:01	C*03:04	C*04:01
B*07:02	C*07:02		C*15:05	
B*53:01	C*04:01	C*06:02	C*12:03	C*02:02
B*41:01	C*17:01	C*07:01	C*16:02	C*06:02
B*58:01	C*03:02	C*07:01	C*07:02	C*06:02
B*35:01	C*04:01		C*15:05	C*16:04
B*18:01	C*12:03	C*07:01	C*02:10	C*15:02
B*49:01	C*07:01		C*04:07	
B*52:01	C*12:02		C*07:02	
B*35:03	C*04:01		C*12:03	
B*14:02	C*08:02		C*06:02	
B*15:17	C*07:01		C*05:01	
B*35:08	C*04:01		C*12:03	
B*13:02	C*06:02		C*16:04	C*18:01
B*38:01	C*12:03		–	
B*15:03	C*04:01	C*02:10	C*07:01	C*12:03
B*07:05	C*15:05		C*07:02	C*06:02
B*44:02	C*05:01	C*16:04	C*07:04	C*02:02

**Table 6:** Number of common (C) and well-documented (WD) alleles (CWD) in 28,927 individuals from the Saudi Stem Cell Donor Registry.

**Locus**	**Number of alleles**	**C**	**WD**	**Total C+WD**	**Non-CWD**	**% CWD**	
A	103	67	2	69	34	67.0	
B	154	93	2	95	59	61.7	
C	76	42	5	47	29	61.8	
DRB1	96	57	3	60	36	62.5	
DQB1	37	22	1	23	14	62.2	
DPB1	74	41	3	44	30	59.5	

### Haplotype Frequencies

A total of 3,430 HLA-A-C-B haplotypes, 1443 HLA-DRB1-DQB1-DPB1 haplotypes, 10,665 HLA-A-C-B-DRB1-DQB1 haplotypes, and 16,394 HLA-A-B-C-DRB1-DQB1-DPB1 haplotypes were estimated. Seven HLA-A~B~DRB1 haplotypes with a frequency more than 1% are shown in Supplementary Table 5. Fourteen HLA-A~C~B haplotypes with a frequency more than 1% are shown in Supplementary Table 6. Twenty-four HLA-DRB1~DQB1~DPB1 haplotypes with a frequency more than 1% are shown in Supplementary Table 7. Seven HLA-A~C~B~DRB1~DQB1 haplotypes are shown in Supplementary Table 8. Thirteen HLA-A~C~B~DRB1~DQB1~DPB1 haplotypes are shown in Table 7 with a frequency >0.5%. Only one HLA-A~C~B~DRB1~DQB1~DPB1 haplotype was of >1% frequency in our population: HLA-A^*^02:01:01G~C^*^15:02:01G~B^*^51:01:01G~DRB1^*^04:02~DQB1^*^03:02:01G~DPB1^*^04:01:0G. A full list of all HLA-A~C~B~DRB1~DQB1 and HLA-A~C~B~DRB1~DQB1~DPB1 haplotypes (observed ≥3 times) is shown in Supplementary Table 9.

**Table 7:** The most Frequent HLA-A~C~B~DRB1~DQB1~DPB1 haplotypes in the Saudi Stem Cell Donor Registry (out of 3,588 haplotypes observed ≥3 times).

**Haplotype**	**Frequency**	**Observed**
A*02:01:01G C*15:02:01G B*51:01:01G DRB1*04:02 DQB1*03:02:01G DPB1*04:01:01G	0.010	611
A*02:01:01G C*07:02:01G B*07:02:01G DRB1*15:01:01G DQB1*06:02:01G DPB1*04:01:01G	0.009	541
A*02:01:01G C*06:02:01G B*50:01:01G DRB1*07:01:01G DQB1*02:01:01G DPB1*04:01:01G	0.008	467
A*23:01:01G C*06:02:01G B*50:01:01G DRB1*07:01:01G DQB1*02:01:01G DPB1*03:01:01G	0.006	386
A*24:02:01G C*07:02:01G B*08:01:01G DRB1*03:01:01G DQB1*02:01:01G DPB1*04:01:01G	0.006	386
A*01:01:01G C*17:01:01G B*41:01 DRB1*07:01:01G DQB1*03:03:02G DPB1*04:02:01G	0.006	372
A*31:01:02G C*15:02:01G B*51:01:01G DRB1*13:01:01G DQB1*06:03:01G DPB1*13:01:01G	0.006	364
A*68:01:01G C*07:02:01G B*08:01:01G DRB1*03:01:01G DQB1*02:01:01G DPB1*04:01:01G	0.006	356
A*02:05:01G C*06:02:01G B*50:01:01G DRB1*07:01:01G DQB1*02:01:01G DPB1*03:01:01G	0.005	347
A*26:01:01G C*07:02:01G B*08:01:01G DRB1*03:01:01G DQB1*02:01:01G DPB1*04:01:01G	0.005	343
A*30:02:01G C*04:01:01G B*53:01:01G DRB1*13:02:01G DQB1*06:09:01G DPB1*04:01:01G	0.005	317
A*02:01:01G C*06:02:01G B*50:01:01G DRB1*07:01:01G DQB1*02:01:01G DPB1*14:01:01G	0.005	305
A*02:05:01G C*06:02:01G B*50:01:01G DRB1*07:01:01G DQB1*02:01:01G DPB1*02:01:02G	0.005	298

For haplotypic diversity, the mean expected heterozygosity was 1.382 (±0.186). In haplotype-level computations, gene diversity and average gene diversity over loci were found to be 0.999 and 0.892 (±0.476), respectively.

### Applications for Matching

**Figure 3:**
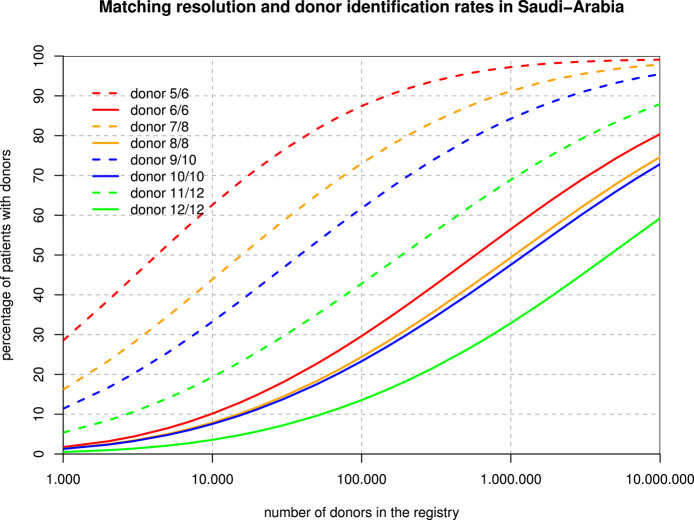
Registry Size and Donor Identification Rates. For each hypothetical registry size, the curves show the fraction of patients finding a fully matched and single mismatched donor when considering 3, 4, 5, and 6 loci.

The 6-locus haplotype frequencies were mapped to P-codes (implicitly ignoring the rare intron-based null alleles), and then, protein-level phenotype frequencies were derived for the purpose of the matching extrapolations. Figure 3 shows that, for example, with one million donors, only about 50% of the patients would find an 8/8 match, but already 90% would get a 7/8 match. Overall, registry sizes required for identical rates of full matches for 3–6 loci are roughly in proportions of 1:2:2:10, and with one mismatch accepted, this ratio is 1:4:10:40.

## Discussion

This is the first report on HLA-A, -B, -C, -DRB1, -DQB1, and -DPB1 alleles and haplotype frequency in a large cohort of Saudis. Our data present 28,927 Saudi individuals from different regions within Saudi Arabia. The allele and haplotype frequencies are similar to what has been reported previously from our donors (17, 18). Seven HLA-A alleles show frequencies higher than 5.0%, including A^*^02:01:01G, A^*^24:02:01G, A^*^01:01:01G, A^*^68:01:01G, A^*^03:01:01G, A^*^31:01:02G, and A^*^26:01:01G. Those alleles represent 57.6% of the allelic diversity observed at this locus.

For HLA-B, only four alleles show a frequency >5%. These alleles are B^*^51:01:01G, B^*^50:01:01G, B^*^08:01:01G, and B^*^07:02:01G. These four alleles account for 43.4% of HLA-B diversity in this cohort. HLA-B^*^50:01 is frequently seen in Arab populations. In Saudi Arabia, B^*^50:01:01 accounts for almost all B^*^50 alleles (19). In this cohort, HLA-B^*^50:01 is the most common B^*^50 allele (seen in 7170 individuals), and only 17 individuals carry the B^*^50:02 allele. Moreover, B^*^50:01:01G is also frequently seen in Caucasians, North Africans, and West-South Asians (20). HLA-B^*^15 exhibits the highest number of alleles as 33 different alleles are detected in this cohort; however, B^*^15 is not a common allele in Saudis. B^*^51 alleles are the second highest polymorphic with 12 different alleles—only B^*^51:01 is the most common. This has implications on finding a matched donor for those individuals carrying the rare B^*^51 or B^*^15 alleles.

For HLA-C, only five alleles show a frequency of >5%. The most frequent HLA-C alleles are C^*^06:02:01G, followed by C^*^07:02:01G, C^*^04:01:01, C^*^152:0, and C^*^07:01:01G. These five alleles account for 61.4% of HLA-C diversity in this cohort. HLA-C^*^07 exhibits the highest number of alleles; 13 different alleles are detected in in this cohort with C^*^07:02 and C^*^07:01 as the most common alleles. In a study comparing serology with molecular typing, we find the same HLA-C common alleles, including HLA-C^*^15, which was not detected by serology (21).

For HLA-DRB1, seven alleles show a frequency of >5%. The most frequent HLA-DRB1 alleles are DRB1^*^DRB1^*^07:01:01G, DRB1^*^03:01:01G, DRB1^*^13:02:01G, DRB1^*^15:01:015G, DRB1^*^13:01:01G, DRB1^*^04:03:0G, and DRB1^*^04:05. These seven alleles account for 59.6% of HLA-DRB1 diversity in this cohort. HLA-DRB1^*^11 and HLA-DRB1^*^14 exhibit the highest number of alleles; 16 different alleles are detected for each of them:15 alleles for HLA-DRB1^*^13 and 13 alleles for HLA-DRB1^*^04. Both HLA-DRB1^*^07:01:01G and DRB1^*^03:01:01G are common in the Central and Eastern regions of Saudi Arabia as previously published (17, 18). In addition, both alleles are frequent in Arabs from Tunisia and Jordan (20). HLA-DRB1^*^13:02:01G is also frequent in some African populations, such as Nigerian and Libyan (20).

For HLA-DQB1, seven alleles showed a frequency of >5%. These alleles include DQB1^*^02:01:01, DQB1^*^03:02:01, DQB1^*^03:01:01, DQB1^*^05:01:01, DQB1^*^06:03:01, DQB1^*^06:02:01G, and DQB1^*^05:02:01. These seven alleles account for 83.9% of HLA-DRB1 diversity in this cohort. HLA-DQB1^*^06 and HLA-DQB1^*^03 exhibit the highest number of alleles; 16 different alleles are detected for DQB1^*^06 and 10 alleles for HLA-DQB1^*^03. HLA-DQB1^*^02:01:01 and DQB1^*^03:01:01 are frequent in Arabs, and HLA-DQB1^*^03:02:01 is frequent in Arabs, Chinese, Indians, and Caucasians (20).

For HLA-DPB1, only five alleles show a frequency of >5%. The most frequent HLA-DPB1 alleles are DPB1^*^04:01:01G, DPB1^*^02:01:02G, DPB1^*^03:01:01, DPB1^*^04:02:01, and DPB1^*^17:01:01. These five alleles account for 80.4% of HLA-DPB1 diversity in this cohort. All top 3 DPB1 alleles are frequent in Asians, especially Chinese, and in Caucasians (20). No single DPB1 type shows diversity; this might be a reflection of the DPB1 nomenclature, which unlike the other HLA genes, does not depend on exon 2. Typing for polymorphisms within DPB1 may not be resolved at the allele level using the current NGS solutions, in which an average fragment size of 200 bp may not resolve cis/trans polymorphisms. This issue, however, can be sorted by third-generation long-read technology (22).

Hardy Weinberg equilibrium analysis shows an excess of homozygotes in this large cohort. This phenomenon was also observed previously by our group (17, 18) and others (23) and might be explained by the high consanguinity marriages in the Saudi population (5). We observed different allele and haplotype frequencies between the central and Eastern provinces of Saudi Arabia (18, 24). Thus, it is important to study different regions of Saudi Arabia independently. However, as with other populations, Saudis are not restricted to their area of origin and many move to Central, Eastern, and Western provinces seeking jobs.

The most common HLA-A~C~B~DRB1~DQB1 haplotype seen in this study is A^*^02:01:01G~C^*^06:02:01G~B^*^50:01:01G~DRB1^*^07:01:01G~DQB1^*^02:01:01G. This haplotype is not common in other populations; however, it is seen at low frequency in American Hispanics, Indian Tamil Nadu, and Columbia cord blood (20). The second most common haplotype is A^*^02:05:01G~C^*^06:02:01G~B^*^50:01:01G~DRB1^*^07:01:01G~DQB1^*^02:01:01G. This haplotype differs from the previous haplotype by the HLA-A^*^02:05 allele and seems to have similar distribution (20). Interesting to note are the common HLA A~C~B~DRB1~DQB1~DPB1 haplotypes; the top three haplotypes account for 3%, and all of them are A^*^02:01:01G-based haplotypes, thus reflecting the high frequency of A^*^02:01:01G, which accounts for 1/5 of the population. These three haplotypes are A^*^02:01:01G~C^*^15:02:01G~B^*^ 51:01:01G~DRB1^*^04:02~DQB1^*^03:02:01G~DPB1^*^04:01:0G, A^*^02:01:01G~C^*^07:02:01G~B^*^07:02:01G~DRB1^*^15:01:01G~ DQB1^*^06:02:01G~DPB1^*^04, and A^*^02:01:01G~C^*^06:02:01G~B^*^50:01:01G~DRB1^*^07:01:01G~DQB1^*^02:01:01G~DPB1^*^04:01:01G. In addition, this result shows that there is strong LD between these alleles with a minimum of crossover events, a phenomenon worth investigating further in the Saudi population. Two locus LD studies reveal that there is a higher frequency of DPB1 association with DRB1 and DQB1, thus reflecting the observation of the extended haplotype across all class I and II gene loci.

HLA null alleles are rare in this population. The following null alleles are found: HLA-C^*^06:49N (two individuals), B^*^37:03N (one individual), A^*^24:312N (one individual), and A^*^30:76N (one individual) (Table 1).

Here, we show the number of CWD alleles. We applied CIWD catalogs 3.0 (8). CWD alleles can be calculated but not intermediate as intermediate alleles require a much larger sample size to be calculated (>1 in 100,000 sample size). CWD alleles for all loci, in our Saudi cohort are in the range of 60%. HLA-B has the highest number of common and highest number of well-documented alleles, and HLA-DQB1 has the lowest numbers for both categories. Our raw data is available through version 3.0 of the CWD catalogs (8).

In any statistical analysis, detail and precision are competing properties, which is reflected in our study by the changing coverage of the gene pool with the significantly positive alleles and haplotypes. Although we are getting more than 99.5% for all individual loci and 97–99% for all two-locus combinations, this is gradually decreasing to 72% for 6-locus haplotypes. We compare that to the German population (12) in which a sample of about 30,000 individuals would cover a very similar part of the polymorphism now well-described based on a sample of several millions.

The different spread between the full match and the one-mismatch curves in Figure 3 is primarily due to the strong linkage disequilibrium between HLA-B and -C as well as between HLA-DRB1 and -DQB1. As a consequence, single mismatches are much less likely than double mismatches. Basically, with a registry size of one million, 10/10 matches might be found for about half of the Arab patients while 60% of patients would find a 9/10 donor among only 100,000 donors. The main caveat in those theoretical calculations is that algorithms and formulae deriving haplotype frequencies from phenotypes and then applying those frequencies back to diploid individuals are all based on the concept of a Hardy Weinberg equilibrium, which our population does not fulfill due to regional subpopulations and non-random mating. On the other hand, it is probably the best extrapolation that can be made on the basis of today's data, and one of the major developments of our century in most regions of the globe will be the abrasion of deviations from HWE in all regions and at all scales.

In conclusion, the results of this study present information that can be used as a tool to identify a hematopoietic stem cell unrelated donor recruitment and selection strategy as well as a helpful tool for population genetic studies and HLA disease associations. Furthermore, knowledge of population-specific allele and haplotype frequency provides hypothetical estimation of the chances of finding matched donors in the registry. There are limitations to our study as we could not stratify our subjects geographically as we have people moving routinely between regions. However, this will be looked at carefully in the future at the time of new donor registration. This may be achieved by asking the donors about the place of birth of both parents and grandparents.

## Data Availability Statement

The original contributions presented in the study are included in the article/Supplementary Material, further inquiries can be directed to the corresponding author/s.

## Ethics Statement

The studies involving human participants were reviewed and approved by Institutional Review Board (IRB), National Guard Health Affairs. Riyadh, Saudi Arabia. The patients/participants provided their written informed consent to participate in this study.

## Author Contributions

AH and DJ: hypothesis and research question. DJ, AA, and AH: research proposal. FU, CM, and AH: data analysis. AH, DJ, CM, and AA: discussing the data. DJ, CM, and AH: writing the manuscript. CM: providing the figures. DJ, FU, AA, CM, and AH: final paper review. All authors: contributed to the writing and analysis of the manuscript.

## Conflict of Interest

The authors declare that the research was conducted in the absence of any commercial or financial relationships that could be construed as a potential conflict of interest. The handling editor declared a past collaboration with the authors.

## References

[1] Marrack P Kappler J The antigen-specific, major histocompatibility complex-restricted receptor on T cells. Adv Immunol. (1986) 38:1–30. 10.1016/S0065-2776(08)60005-X 3083653

[2] Robinson J Barker DJ Georgiou X Cooper MA Flicek P Marsh SGE. IPD-IMGT/HLA database. Nucleic Acids Res. (2020) 48:D948–55. 10.1093/nar/gkz950 31667505 PMC7145640

[3] Dehn J Arora M Spellman S Setterholm M Horowitz M Confer D. Unrelated donor hematopoietic cell transplantation: factors associated with a better HLA match. Biol Blood Marrow Transplant. (2008) 14:1334–40. 10.1016/j.bbmt.2008.09.009 19041054 PMC3319684

[4] Shiina T Inoko H Kulski JK. An update of the HLA genomic region, locus information and disease associations: 2004. Tissue Antigens. (2004) 64:631–49. 10.1111/j.1399-0039.2004.00327.x 15546336

[5] el-Hazmi MA al-Swailem AR Warsy AS al-Swailem AM Sulaimani R al-Meshari AA. Consanguinity among the Saudi Arabian population. J Med Genet. (1995) 32:623–6. 10.1136/jmg.32.8.623 7473654 PMC1051637

[6] Jawdat DM Al Saleh S Sutton P Al Anazi H Shubaili A Tamim H. Chances of finding an HLA-matched sibling: the Saudi experience. Biol Blood Marrow Transplant. (2009) 15:1342–4. 10.1016/j.bbmt.2009.06.013 19747644

[7] Cereb N Kim HR Ryu J Yang SY. Advances in DNA sequencing technologies for high resolution HLA typing. Hum Immunol. (2015) 76:923–7. 10.1016/j.humimm.2015.09.015 26423536

[8] Hurley CK Kempenich J Wadsworth K Sauter J Hofmann JA Hofmann D. Common, intermediate and well-documented HLA alleles in world populations: CIWD version 3.0.0. HLA. (2020) 95:516–31. 10.1111/tan.13811 31970929 PMC7317522

[9] Excoffier L Laval G Schneider S. Arlequin (version 3.0): an integrated software package for population genetics data analysis. Evol Bioinform Online. (2007) 1:47–50. 10.1177/117693430500100003 19325852 PMC2658868

[10] Niu T. Algorithms for inferring haplotypes. Genet Epidemiol. (2004) 27:334–47. 10.1002/gepi.20024 15368348

[11] Sonnenberg FA Eckman MH Pauker SG. Bone marrow donor registries: the relation between registry size and probability of finding complete and partial matches. Blood. (1989) 74:2569–78. 10.1182/blood.V74.7.2569.2569 2804379

[12] Eberhard HP Muller CR. The impact of HLA-C matching on donor identification rates in a european-caucasian population. Front Immunol. (2014) 5:501. 10.3389/fimmu.2014.00501 25360136 PMC4197773

[13] Dehn J Spellman S Hurley CK Shaw BE Barker JN Burns LJ. Selection of unrelated donors and cord blood units for hematopoietic cell transplantation: guidelines from the NMDP/CIBMTR. Blood. (2019) 134:924–34. 10.1182/blood.2019001212 31292117 PMC6753623

[14] Fakhoury HA Jawdat D Alaskar AS Al Jumah M Cereb N Hajeer AH. Two novel alleles HLA-A^*^02:433 and HLA-A^*^02:434 identified in Saudi bone marrow donors using sequence-based typing. Int J Immunogenet. (2014) 41:338–9. 10.1111/iji.12131 24919814

[15] Fakhoury HA Cereb N Jawdat D Al Jumah M Alaskar AS Hajeer AH. Two novel alleles HLA-DRB1^*^11:150 and HLA-DRB1^*^14:145 identified in Saudi individuals. Int J Immunogenet. (2014) 41:340–1. 10.1111/iji.12133 24920052

[16] Fakhoury HA Jawdat D Alaskar AS Al Jumah M Cereb N Hajeer AH. Three new HLA-C alleles (HLA-C^*^14:02:13, HLA-C^*^15:72 and HLA-C^*^15:74) in Saudi bone marrow donors. Int J Immunogenet. (2015) 42:359–60. 10.1111/iji.12218 26239392

[17] Askar M Daghstani J Thomas D Leahy N Dunn P Claas F. 16th IHIW: global distribution of extended HLA haplotypes. Int J Immunogenet. (2013) 40:31–8. 10.1111/iji.12029 23302097

[18] Jawdat D Al-Zahrani M Al-Askar A Fakhoury H Uyar FA Hajeer A. HLA-A, B, C, DRB1 and DQB1 allele and haplotype frequencies in volunteer bone marrow donors from Eastern Region of Saudi Arabia. HLA. (2019) 94:49–56. 10.1111/tan.13533 30903680

[19] Jawdat D Al-Hamad B Al-Jumah M Hajeer A. HLA-B50 polymorphism in the Saudi population. Int J Immunogenet. (2014) 41:95–7. 10.1111/iji.12096 24256064

[20] Gonzalez-Galarza FF McCabe A Melo Dos Santos EJ Takeshita L Ghattaoraya G Jones AR. Allele frequency net database. Methods Mol Biol. (2018) 1802:49–62. 10.1007/978-1-4939-8546-3_4 29858801

[21] Jawdat D Al Saleh S Sutton P Anazi AL Shubaili A Uyar FY. HLA-C polymorphisms in two cohorts of donors for bone marrow transplantation. Saudi J Kidney Dis Transpl. (2012) 23:467–70. 22569429

[22] Klasberg S Lang K Gunther M Schober G Massalski C Schmidt AH. Patterns of non-ARD variation in more than 300 full-length HLA-DPB1 alleles. Hum Immunol. (2019) 80:44–52. 10.1016/j.humimm.2018.05.006 29879452

[23] Ollier W Doyle P Alonso A Awad J Williams E Gill D. HLA polymorphisms in Saudi Arabs. Tissue Antigens. (1985) 25:87–95. 10.1111/j.1399-0039.1985.tb00420.x 3857723

[24] Hajeer AH Al Balwi MA Uyar AF Al Jumah MA HLA-A, -B, -C, -DRB1, -DQA1, -DQB1 AND -DPB1 allele frequencies in a Saudi population using next generation sequencing technique. Human Immunol. (2013) 74:146 10.1016/j.humimm.2013.08.214 24461004

