# Supplementary material for: HLA-A, -B, -C, -DRB1, -DQB1, and -DPB1 Allele and Haplotype Frequencies of 28,927 Saudi Stem Cell Donors Typed by Next-Generation Sequencing
Source: Front Immunol. 2020 Oct 22;11:544768. doi: 10.3389/fimmu.2020.544768 (PMC7643328; doi:10.3389/fimmu.2020.544768)

Figure S1A: The haplotypes of each locus pair are ordered by descending frequency on the x-axis and the curve shows their frequency. Both axes are in logarithmic scale. The dotted horizontal line shows the frequency corresponding to three copies in the sample.

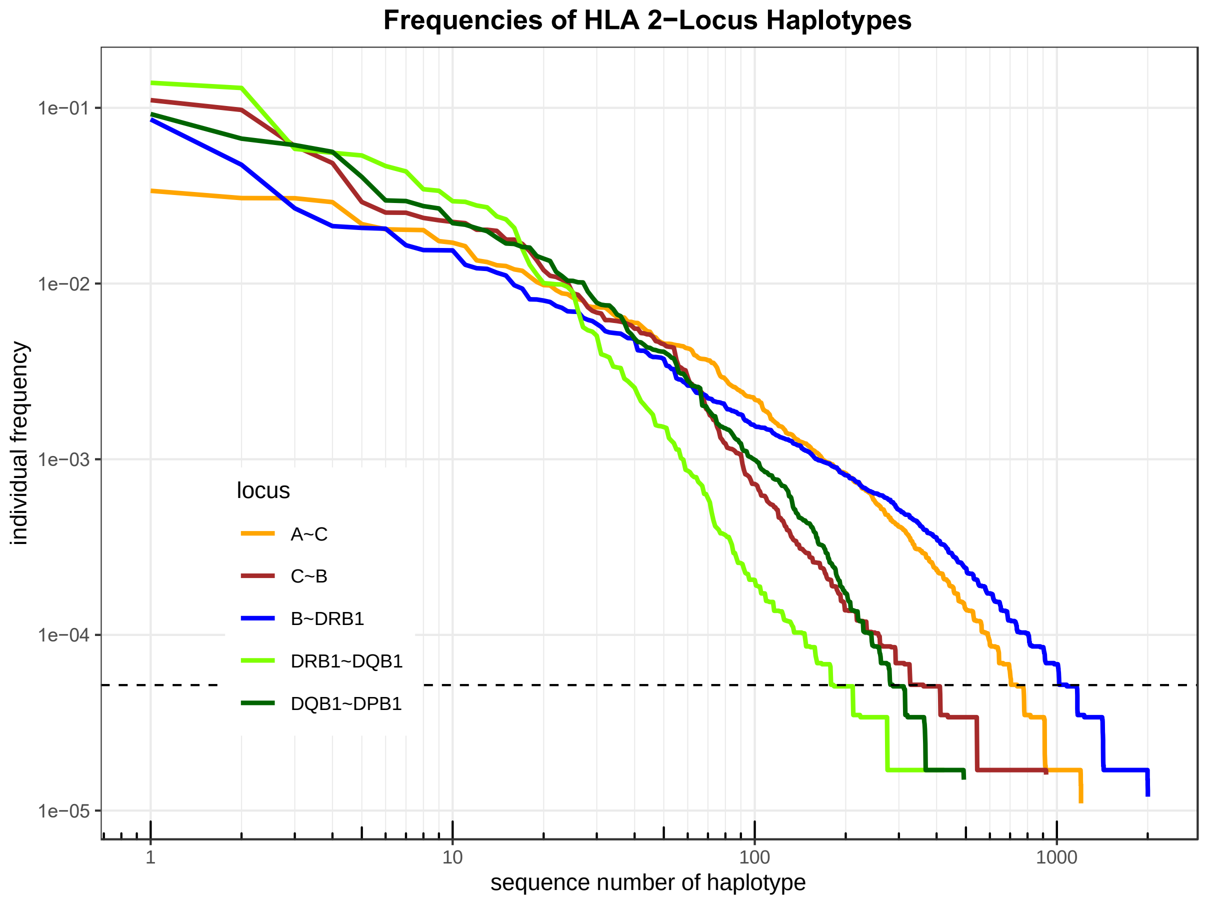

Figure S1B: For each number n on the x-axis in logarithmic scale, the curve depicts the cumulative frequency of the set of the n most frequent haplotypes of each locus combination. The dots on each curve mark the cumulative frequency of all haplotypes seen at least three times in the sample.

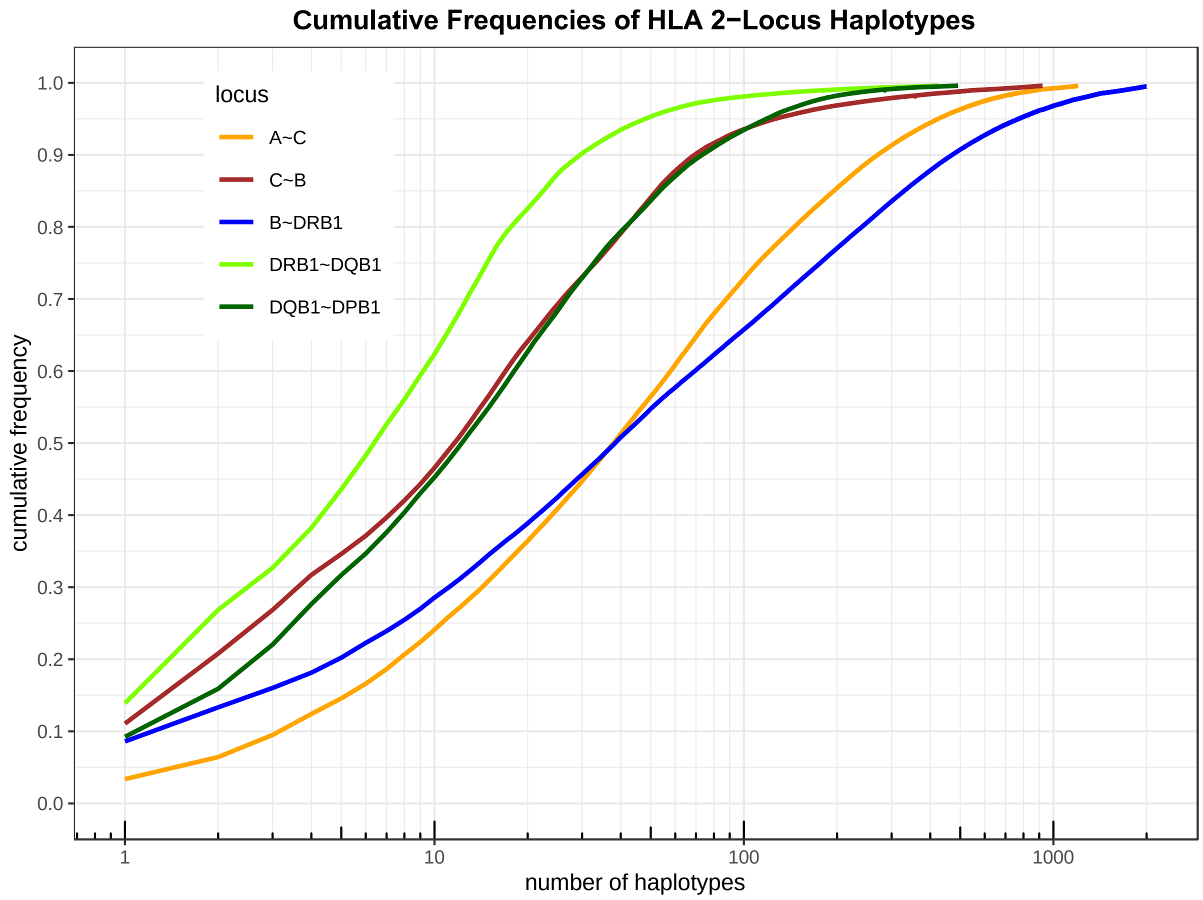

Figure S2A: The haplotypes of each locus pair are ordered by descending frequency on the x-axis and the curve shows their frequency. Both axes are in logarithmic scale. The dotted horizontal line shows the frequency corresponding to three copies in the sample.

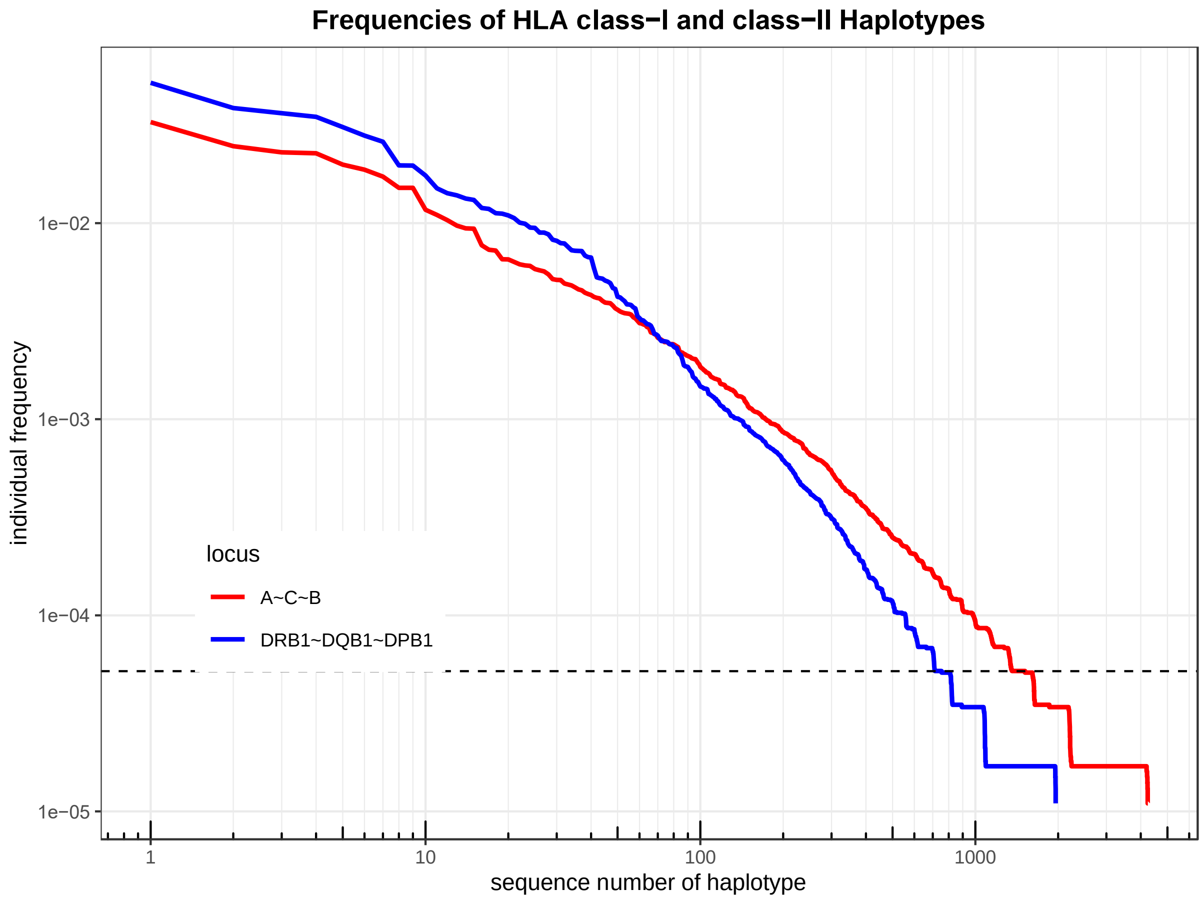

Figure S2B: For each number n on the x-axis in logarithmic scale, the curve depicts the cumulative frequency of the set of the n most frequent haplotypes of each locus combination. The dots on each curve mark the cumulative frequency of all haplotypes seen at least three times in the sample.

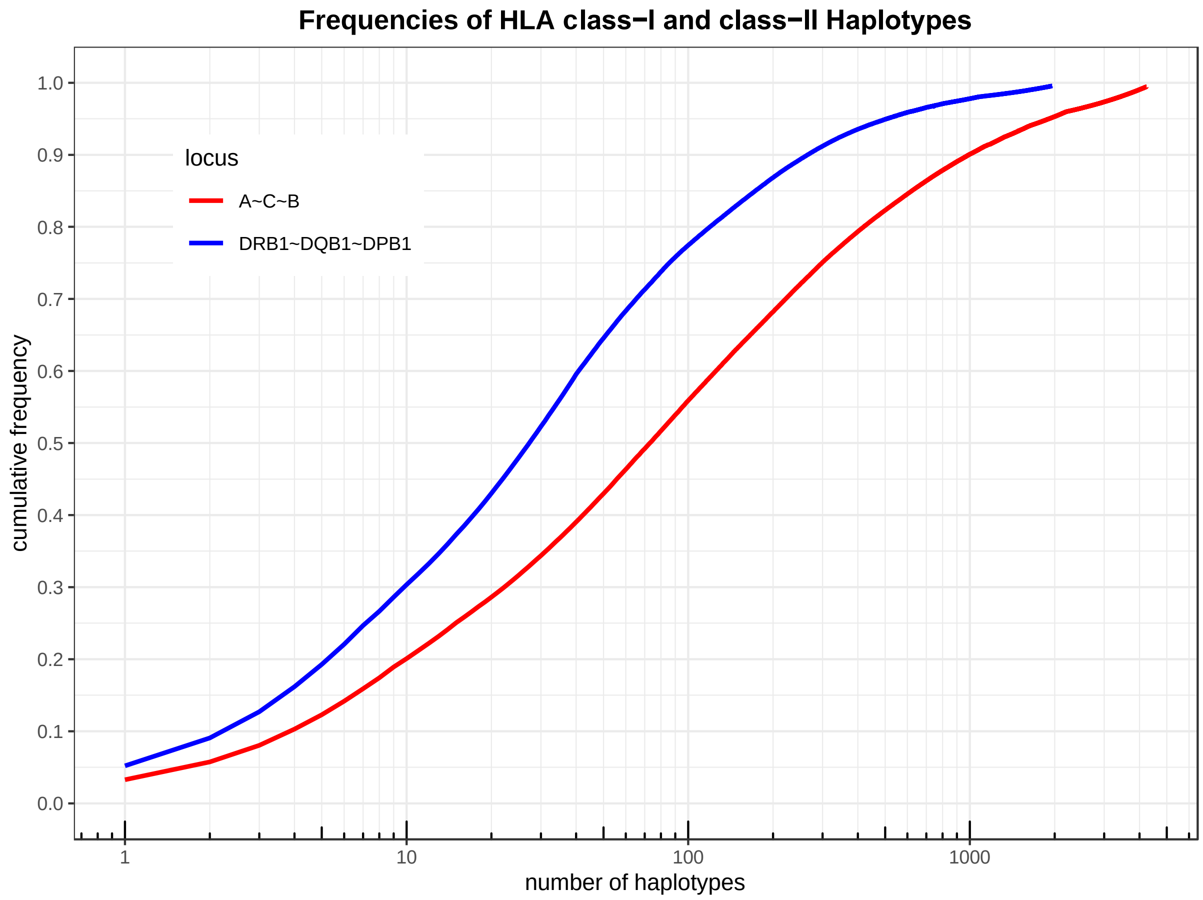

Supplement: Supplementary file 1 [file Data_Sheet_1.PDF]
