# Supplementary material for: HLA-A, -B, -C, -DRB1, -DQB1, and -DPB1 Allele and Haplotype Frequencies of 28,927 Saudi Stem Cell Donors Typed by Next-Generation Sequencing
Source: Front Immunol. 2020 Oct 22;11:544768. doi: 10.3389/fimmu.2020.544768 (PMC7643328; doi:10.3389/fimmu.2020.544768)
Supplement: Supplementary file 2 [file Data_Sheet_2.zip › Supplementary Table S1-S4.DOCX]

**Table S1.** Two locus combination haplotype cumulated frequency. For the haplotype frequencies of all 5 adjacent two-locus pairs, the table shows Shannon’s entropy of the frequency distribution as well as the number of the most frequent haplotypes required to cover 50 %, 75 %, 90 % and 95 % of the gene pool as well as the total number of haplotypes observed in the sample

|  | **Shannon's** | | **haplotypes required to cover** | | | |  |
| --- | --- | --- | --- | --- | --- | --- | --- |
| **Locus combination** | | **entropy** | **50%** | **75%** | **90%** | **95%** | **total** |
| A~C | | 7.74 | 38 | 111 | 271 | 425 | 1204 |
| C~B | | 5.99 | 12 | 34 | 70 | 127 | 922 |
| B~DRB1 | | 8.03 | 39 | 177 | 471 | 773 | 2002 |
| DRB1~DQB1 | | 4.95 | 7 | 15 | 30 | 49 | 424 |
| DQB1~DPB1 | | 6.03 | 13 | 33 | 74 | 119 | 493 |

**Table S2.** Class I and class II haplotypes cumulated frequency. For the haplotype frequencies, the table shows Shannon’s entropy of the frequency distribution as well as the number of the most frequent haplotypes required to cover 50 %, 75 %, 90 % and 95 % of the gene pool as well as the total number of haplotypes observed in the sample.

|  | **Shannon's** | **haplotypes required to cover** | | | | |  |
| --- | --- | --- | --- | --- | --- | --- | --- |
| **locus combination** | **entropy** | **50%** | **75%** | **90%** | **95%** | **total** | |
| A~C~B | 9.03 | 74 | 299 | 991 | 1871 | 4249 | |
| DRB1~DQB1~DPB1 | 7.50 | 28 | 86 | 266 | 507 | 1961 | |

**Table S3.** For the haplotype frequencies of all 5 sets of loci frequently used in matching, the table shows Shannon’s entropy of the frequency distribution as well as the number of the most frequent haplotypes required to cover 50 %, 75 %, 90 % and 95 % of the gene pool as well as the total number of haplotypes observed in the sample.

|  | **Shannon's** | **haplotypes required to cover** | | |  |  |
| --- | --- | --- | --- | --- | --- | --- |
| **locus combination** | **entropy** | **50%** | **75%** | **90%** | **95%** | **total** |
| A~B | 8.27 | 51 | 183 | 489 | 806 | 2111 |
| A~B~DRB1 | 10.63 | 224 | 1252 | 3883 | 6389 | 9125 |
| A~C~B~DRB1 | 10.96 | 286 | 1683 | 5434 | 8375 | 11127 |
| A~C~B~DRB1~DQB1 | 11.05 | 303 | 1835 | 6128 | 9069 | 11826 |
| A~C~B~DRB1~DQB1~DPB1 | 12.08 | 752 | 3964 | 10621 | 13562 | 16394 |
