# Supplementary material for: HLA-A, -B, -C, -DRB1, -DQB1, and -DPB1 Allele and Haplotype Frequencies of 28,927 Saudi Stem Cell Donors Typed by Next-Generation Sequencing
Source: Front Immunol. 2020 Oct 22;11:544768. doi: 10.3389/fimmu.2020.544768 (PMC7643328; doi:10.3389/fimmu.2020.544768)
Supplement: Supplementary file 2 [file Data_Sheet_2.zip › Supplementary Table S6.DOCX]

Table S6: Frequent HLA- A~C~B haplotypes (frequency > 0.01) in the Saudi Stem Cell Donor Registry.

| Haplotype | Frequency |
| --- | --- |
| A*02:01:01G C*15:02:01G B*51:01:01G | 0.032 |
| A*02:01:01G C*06:02:01G B*50:01:01G | 0.024 |
| A*02:05:01G C*06:02:01G B*50:01:01G | 0.023 |
| A*02:01:01G C*07:02:01G B*07:02:01G | 0.022 |
| A*31:01:02G C*15:02:01G B*51:01:01G | 0.020 |
| A*23:01:01G C*06:02:01G B*50:01:01G | 0.019 |
| A*26:01:01G C*07:02:01G B*08:01:01G | 0.017 |
| A*30:02:01G C*04:01:01G B*53:01:01G | 0.015 |
| A*68:01:01G C*07:02:01G B*08:01:01G | 0.015 |
| A*02:01:01G C*14:02:01G B*51:01:01G | 0.011 |
| A*01:01:01G C*17:01:01G B*41:01 | 0.011 |
| A*24:02:01G C*07:02:01G B*08:01:01G | 0.010 |
| A*33:03:01G C*03:02:01G B*58:01:01G | 0.010 |
| A*02:01:01G C*16:02:01G B*51:01:01G | 0.010 |
