# Supplementary material for: HLA-A, -B, -C, -DRB1, -DQB1, and -DPB1 Allele and Haplotype Frequencies of 28,927 Saudi Stem Cell Donors Typed by Next-Generation Sequencing
Source: Front Immunol. 2020 Oct 22;11:544768. doi: 10.3389/fimmu.2020.544768 (PMC7643328; doi:10.3389/fimmu.2020.544768)
Supplement: Supplementary file 2 [file Data_Sheet_2.zip › Supplementary Table S7.DOCX]

Table S7: Frequent HLA- DRB1~DQB1~DPB1 haplotypes (frequency > 0.01) in the Saudi Stem Cell Donor Registry.

| **Haplotype** | **Frequency** |
| --- | --- |
| DRB1*03:01:01G DQB1*02:01:01G DPB1*04:01:01G | 0.050 |
| DRB1*07:01:01G DQB1*02:01:01G DPB1*03:01:01G | 0.039 |
| DRB1*04:03:01G DQB1*03:02:01G DPB1*04:01:01G | 0.038 |
| DRB1*07:01:01G DQB1*02:01:01G DPB1*04:01:01G | 0.033 |
| DRB1*03:01:01G DQB1*02:01:01G DPB1*02:01:02G | 0.030 |
| DRB1*15:01:01G DQB1*06:02:01G DPB1*04:01:01G | 0.029 |
| DRB1*07:01:01G DQB1*02:01:01G DPB1*02:01:02G | 0.024 |
| DRB1*11:01:01G DQB1*03:01:01G DPB1*04:01:01G | 0.020 |
| DRB1*13:02:01G DQB1*06:04:01G DPB1*04:01:01G | 0.020 |
| DRB1*04:02 DQB1*03:02:01G DPB1*04:01:01G | 0.017 |
| DRB1*13:01:01G DQB1*06:03:01G DPB1*04:01:01G | 0.016 |
| DRB1*13:02:01G DQB1*06:04:01G DPB1*02:01:02G | 0.014 |
| DRB1*13:01:01G DQB1*06:03:01G DPB1*02:01:02G | 0.014 |
| DRB1*15:01:01G DQB1*06:02:01G DPB1*02:01:02G | 0.014 |
| DRB1*03:01:01G DQB1*02:01:01G DPB1*03:01:01G | 0.013 |
| DRB1*15:02:01G DQB1*06:01:01G DPB1*04:01:01G | 0.012 |
| DRB1*07:01:01G DQB1*02:01:01G DPB1*14:01:01G | 0.012 |
| DRB1*16:01 DQB1*05:02:01G DPB1*02:01:02G | 0.011 |
| DRB1*07:01:01G DQB1*02:01:01G DPB1*17:01:01G | 0.011 |
| DRB1*10:01:01G DQB1*05:01:01G DPB1*04:01:01G | 0.011 |
| DRB1*13:01:01G DQB1*06:03:01G DPB1*13:01:01G | 0.010 |
| DRB1*04:03:01G DQB1*03:02:01G DPB1*02:01:02G | 0.010 |
| DRB1*13:02:01G DQB1*06:09:01G DPB1*04:01:01G | 0.010 |
| DRB1*10:01:01G DQB1*05:01:01G DPB1*02:01:02G | 0.010 |
