# Supplementary material for: HLA-A, -B, -C, -DRB1, -DQB1, and -DPB1 Allele and Haplotype Frequencies of 28,927 Saudi Stem Cell Donors Typed by Next-Generation Sequencing
Source: Front Immunol. 2020 Oct 22;11:544768. doi: 10.3389/fimmu.2020.544768 (PMC7643328; doi:10.3389/fimmu.2020.544768)
Supplement: Supplementary file 2 [file Data_Sheet_2.zip › Supplementary Table S8.DOCX]

Table S8: Frequent HLA- A~C~B~DRB1~DQB1 haplotypes (frequency > 0.01) in the Saudi Stem Cell Donor Registry.

| Haplotype | Frequency |
| --- | --- |
| A*02:01:01G C*06:02:01G B*50:01:01G DRB1*07:01:01G DQB1*02:01:01G | 0.019 |
| A*02:05:01G C*06:02:01G B*50:01:01G DRB1*07:01:01G DQB1*02:01:01G | 0.016 |
| A*26:01:01G C*07:02:01G B*08:01:01G DRB1*03:01:01G DQB1*02:01:01G | 0.015 |
| A*31:01:02G C*15:02:01G B*51:01:01G DRB1*13:01:01G DQB1*06:03:01G | 0.014 |
| A*02:01:01G C*15:02:01G B*51:01:01G DRB1*04:02 DQB1*03:02:01G | 0.014 |
| A*23:01:01G C*06:02:01G B*50:01:01G DRB1*07:01:01G DQB1*02:01:01G | 0.012 |
| A*02:01:01G C*07:02:01G B*07:02:01G DRB1*15:01:01G DQB1*06:02:01G | 0.012 |
